# A Clinical Algorithm for Full‐Mouth Rehabilitation in Patients With Generalized Severe Tooth Wear: A Case Report

**DOI:** 10.1002/cre2.70349

**Published:** 2026-04-26

**Authors:** Łukasz Lassmann, Adam Pióro, Javier Calatrava, Hom‐Lay Wang

**Affiliations:** ^1^ Private Practice, One & Only Clinic Gdańsk Poland; ^2^ Lassmann Education Center Gdańsk Poland; ^3^ Department of Periodontics and Oral Medicine University of Michigan School of Dentistry Ann Arbor Michigan USA; ^4^ Section of Graduate Periodontology University Complutense Madrid Spain

**Keywords:** erosion, ferrule, sds concept, smile design, TMD, tooth wear, vertical dimension of occlusion

## Abstract

**Objectives:**

To present a structured decision‐making approach to full‐mouth rehabilitation in patients with generalized severe tooth wear, using functional risk classification (Green, Yellow, Red) and the Smile Design and Space (SDS) Concept for vertical dimension planning.

**Material and Methods:**

A 65‐year‐old male presented with generalized erosion and attrition, affecting esthetics and speech. A structured diagnostic protocol began with symptom‐based questionnaires and clinical examination. He was categorized as “Yellow” indicating the absence of temporomandibular disorders (TMD), repeatable occlusion, but need for interdisciplinary pretreatment. The SDS Concept guided the vertical dimension of occlusion (VDO) increase, based on incisal display, smile line, and prosthetic space, using the formula A + B − C = VDO. Functional crown lengthening was performed to restore the ferrule. Restorations were done with adhesively bonded lithium disilicate crowns and layered ceramics.

**Results:**

A 10 mm VDO increase was well tolerated, with restored esthetics, stable occlusion, and improved phonetics. The SDS protocol provided a reproducible, esthetically driven method for VDO and prosthetic space evaluation with no complications.

**Conclusions:**

Integrating functional risk classification with the SDS concept enables individualized, systematic full‐mouth rehabilitation in severe tooth wear cases, improving diagnostic precision and interdisciplinary communication.

## 1 Introduction

Managing patients with severe tooth wear poses significant diagnostic and therapeutic challenges in restorative dentistry. The underlying etiology of wear is often multifactorial, involving mechanical forces such as attrition and abrasion, as well as chemical erosion from dietary or systemic sources (Bartlett and O'Toole 2021; Loomans et al. 2017; Carvalho et al. 2015). Compounding the difficulty, in many cases, the exact cause of tooth surface loss remains unclear at the time of clinical presentation. As a result, treatment success depends not only on restoring form and function but also on correctly identifying predisposing risk factors and determining whether the case is suitable for immediate prosthetic intervention or requires preliminary management.

The controversy surrounding changes in the vertical dimension of occlusion (VDO) persists, largely fueled by a mix of anecdotal beliefs and the diverse array of techniques described in the literature, ranging from conventional facial reference points to advanced digital tools (Lassmann, Calamita, and Manfredini 2025). Rather than being guided by functional outcomes or scientific validation, the choice of method is often shaped by clinical habit and practical convenience (Manfredini et al. 2023).

To address these challenges, a structured diagnostic workflow combining functional risk stratification and esthetic–spatial analysis is proposed and described in detail in the Methods section.

**Figure 1:**
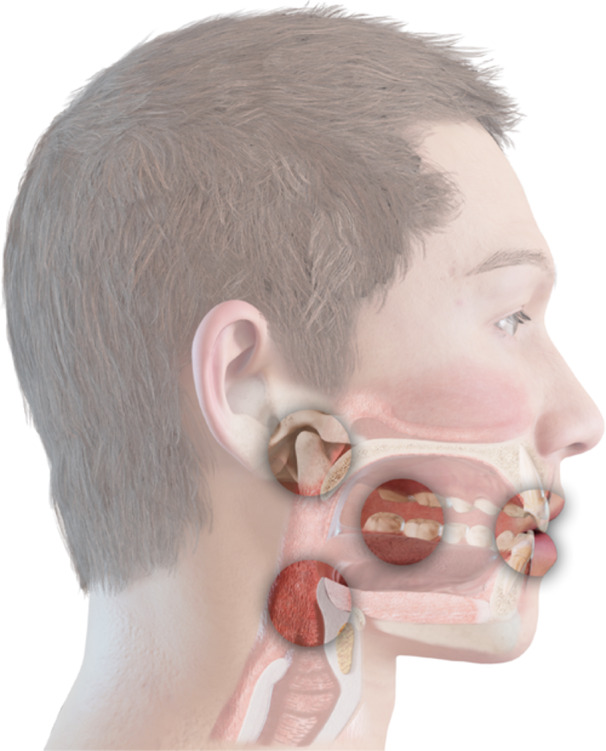
In the SDS Concept, “space” refers to four distinct anatomical zones that can be evaluated and managed during full‐mouth rehabilitation: the anterior restorative space, the posterior occlusal space, the TMJ seating space, and the airway space.

While this clinical stratification provides a clear roadmap for evaluating functional risk, it does not address the esthetic and spatial implications of managing vertical dimension: an essential component in cases of advanced wear. For this reason, the diagnostic process incorporates a second algorithm, known as the Smile Design and Space (SDS) Concept (Lassmann, Calamita, and Blatz 2025). This system integrates fundamental principles of Digital Smile Design (Coachman and Marcelo 2012) and Facially Generated Treatment Planning (FGTP) (Spear 2016; Spear and Kokich 2007; Hempton and Dominici 2010) with the coordinated management of four interrelated functional spaces: the anterior restorative zone, the posterior occlusal space, the condylar seating space within the temporomandibular joints, and the airway space (Figure 1).

**Figure 2:**
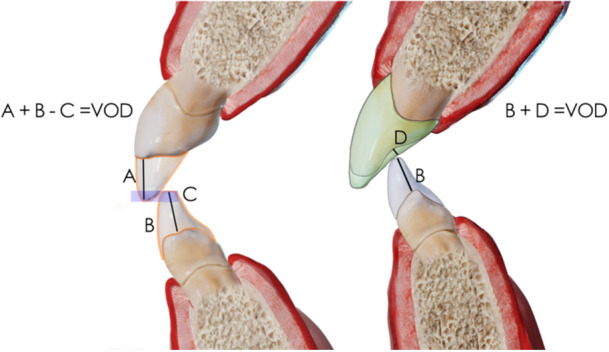
The required increase in vertical dimension of occlusion can be calculated using a formula where: A is the planned elongation of the upper incisors, B is the elongation of the lower incisors, C is the intended overbite, and D is the palatal morphology. The formula used depends on the incisal relationship.

The SDS concept introduces quantitative precision into VDO planning, employing novel formulas (Figure 2). In situations where the worn incisors present with an edge‐to‐edge relationship, elongating the incisal edges is not feasible without either increasing the VDO or performing orthodontic intrusion. To reestablish an edge‐to‐edge relationship with elongated incisors, the required VDO increase corresponds to the combined length of the upper and lower incisor elongation, represented by the equation A + B = VDO (Figure 2). However, if the goal is to preserve a minimal vertical overbite of 2 mm for functional purposes, this value must be subtracted from the total, resulting in the refined formula: A + B − C = VDO (Figure 2).

Conversely, in Angle Class I or II incisal relationships, where incisal relationships involve the upper incisors overlapping the lower incisors, lengthening the upper incisors does not directly influence the VDO; care must be taken to avoid interference between the palatal surface of the maxillary incisors and the incisal edges of the mandibular teeth. In such cases, the vertical space required to accommodate posterior restorations or anterior guidance is calculated by adding the elongation of the lower incisors to the desired palatal clearance, using the formula B + D = VDO (Figure 2).

When integrated into a unified protocol, these two algorithms, functional risk classification and esthetic‐spatial assessment, provide a robust foundation for predictable treatment. In this article, we discuss a full‐mouth rehabilitation in a patient with generalized tooth wear and no history of pain, managed entirely using this dual framework. By combining interdisciplinary screening, detailed questionnaire analysis, and SDS‐guided VDO planning, this protocol achieved a stable, esthetic, and functionally sound outcome. A 2‐year follow‐up confirms the longevity and effectiveness of this structured, decision‐based approach to complex prosthetic rehabilitation.

While multiple approaches to managing worn dentitions have been described, there is a lack of structured, clinically applicable algorithms that simultaneously address functional risk, esthetic planning, and vertical dimension management within a single workflow. This article aims to bridge this gap by presenting a dual‐algorithmic approach illustrated through a clinical case.

## 2 Materials and Methods—Technique Description

The proposed decision‐making process for full‐mouth rehabilitation in patients with severe tooth wear is based on a structured diagnostic algorithm that combines clinical assessment, imaging, and comprehensive symptom‐guided questionnaires. The objective was to identify both the etiology of tooth wear and any risk factors that could compromise prosthetic outcomes if left unaddressed.

### 2.1 Initial Screening and Questionnaire Workflow

**Figure 3:**
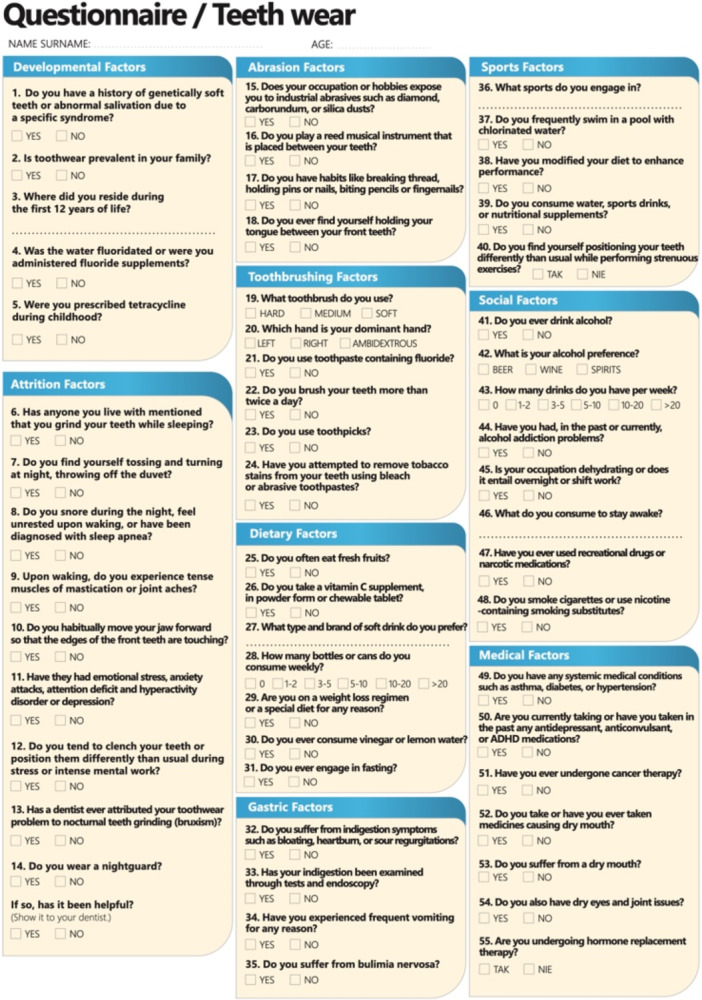
Teeth wear multifactorial screening questionnaire.

During the initial visit, patients are guided through a structured questionnaire system to identify relevant symptoms and direct them to the appropriate next steps. This system ensures:

- *Sleep‐related disorders*: Patients suspected of having sleep disorders complete an “Introductory Questionnaire” to assess the risk of apnea, snoring, daytime fatigue, and parafunctional behaviors like clenching or grinding during sleep. This is crucial due to the link between bruxism and obstructive sleep apnea (OSA), as well as the potential exacerbation of OSA after an increase in VDO (Ye Min Soe et al. 2022; Gagnon et al. 2004; Nikolopoulou et al. 2013, 2011; Vroegop et al. 2012).
- *Unexplained tooth wear*: Patients with unexplained tooth wear fill out a “Tooth Wear Etiology Questionnaire” (Figure 3) which inquiries about diet (e.g., acidic beverages, citrus intake), gastrointestinal health, stress patterns, chewing habits, medication use, and occupational risk factors.
- *Pain or joint‐related symptoms*: Patients reporting pain or joint symptoms complete a “TMD and Orofacial Pain Questionnaire,” which addresses pain characteristics, bruxism, joint and muscle symptoms, lifestyle factors, and treatment history.

### 2.2 Data Collection and Clinical Examination

After completing the questionnaires, patients undergo a comprehensive clinical examination which includes:

- *Intraoral and extraoral evaluation*: This focuses on assessing the extent and pattern of tooth wear, caries, occlusion, soft tissue characteristics, gingival recession, and periodontal health.
- *Temporomandibular joint (TMJ) evaluation*: This involves palpation, assessing range of motion, pain mapping, and checking for deviation or deflection during opening, as well as auscultating for joint sounds.
- *Functional assessment*: This includes testing the repeatability of centric relation with a leaf gauge, palpation of muscles, and identification of occlusal instability.
- *Radiographic imaging*: Cone beam computed tomography (CBCT) is utilized to evaluate periapical status, bone quality, TMJ integrity, and sinus involvement. Three‐dimensional planning tools, such as an intraoral scan (iOS), are used when surgical crown lengthening or implant therapy is anticipated.

### 2.3 Algorithmic Classification

**Figure 4:**
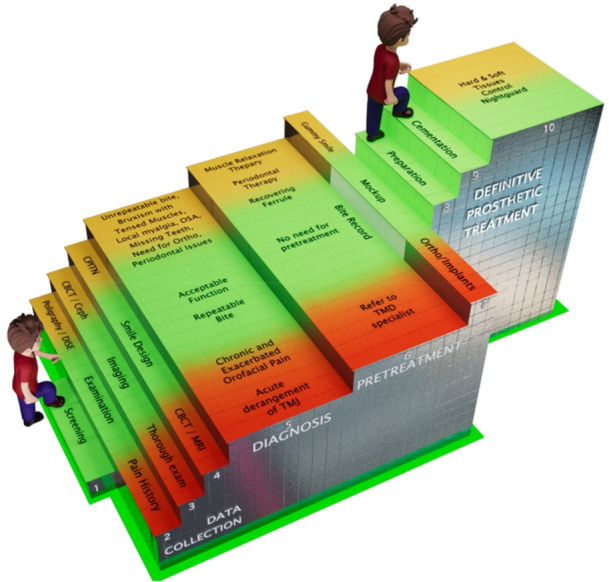
Diagnostic decision algorithm, categorizing patients with severe tooth wear into Green, Yellow, or Red groups based on: presence of pain, occlusal stability, and need for interdisciplinary pretreatment.

Based on the diagnostic findings, patients are categorized into three clinical groups:

- *Green category*: Patients without TMD symptoms, with stable and repeatable occlusion, and no need for significant interdisciplinary intervention. They can proceed directly with prosthetic planning, with potential VDO increases using a leaf gauge.
- *Yellow category*: Patients with moderate complexity, such as unrepeatable bites, signs of parafunction, periodontal issues, esthetic gingival asymmetry, mild sleep apnea, or orthodontic needs. Pretreatment may involve occlusal splints, physiotherapy, periodontal or surgical procedures, or orthodontics before prosthetic therapy.
- *Red category*: Patients with active TMJ degeneration or chronic, centrally mediated orofacial pain. They require interdisciplinary management, including consultations with pain specialists, sleep medicine professionals, psychologists, or rheumatologists, prior to any restorative intervention (Figure 4).

### 2.4 Three‐Step Algorithm Core

**Figure 5:**
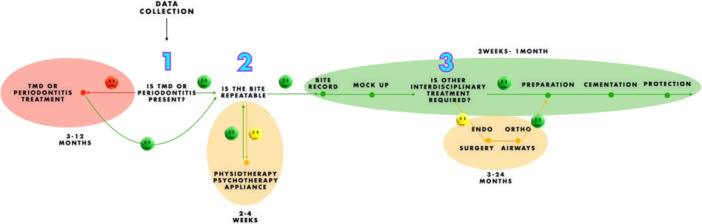
Three‐step decision algorithm guiding full‐mouth rehabilitation: (1) TMD or periodontitis assessment, (2) bite repeatability, and (3) need for interdisciplinary treatment. Only stable cases may proceed to final prosthetic planning.

The core diagnostic pathway consists of three key clinical questions that guide the decision‐making process (Figure 5):

1. Is there orofacial pain/TMD or periodontitis present?
  ◦ Yes: Treat or refer before proceeding.
  ◦ No: Move to Step 2.

2. Is the bite stable and repeatable?
  ◦ Yes: Proceed to Step 3.
  ◦ No: Initiate pretreatment to achieve stability.

3. Is any other interdisciplinary treatment needed before prosthetic therapy?
  ◦ Yes: Coordinate referrals and complete the necessary pretreatments.
  ◦ No: Proceed to definitive prosthetic planning.

### 2.5 SDS Concept Integration

After achieving functional readiness, the SDS concept facilitates the precise planning of vertical dimension increase, tailored to both esthetic requirements and biomechanical constraints. This approach ensures that restorative design supports both visual harmony and long‐term functional balance.

## 3 Results—Case Report

**Figure 6:**
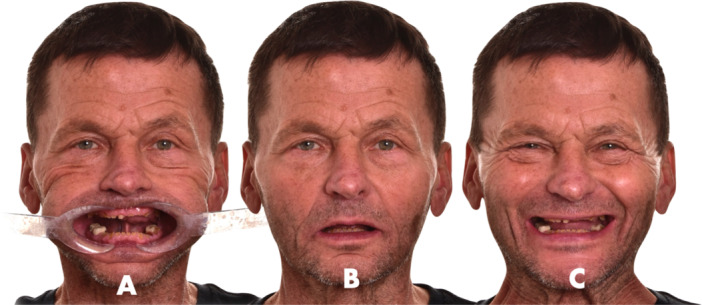
For accurate smile design in patients with advanced tooth wear, three standardized photographs are essential: one with retractors, one in the resting lip position, and one at full smile. Consistent head positioning across all images is crucial, as it enables precise overlay using basic tools like PowerPoint or Keynote by adjusting transparency. This enables clinicians to visualize incisal display, smile line, and restorative space within a single visual reference.

A 65‐year‐old male presented with the chief complaint of deteriorating speech articulation and smile esthetics affecting his professional communication. He reported no systemic conditions, TMJ pain, or sleep‐related issues. He was systemically healthy, maintaining a healthy lifestyle with regular physical activity and strict oral hygiene (Figure 6). The patient was informed about the documentation of the case to be published, and agreed to sign an informed consent form.

The wear etiology was initially unclear, as he denied harmful habits or gastric reflux. However, a detailed teeth wear questionnaire revealed several contributing factors: frequent intake of chewable vitamin C supplements (Giunta 1983; Bahal and Djemal 2014) extended fasting periods leading to reduced salivary buffering capacity (Johansson et al. 1984), and jaw clenching during combat sports due to stress (Polmann et al. 2020). These findings led to a diagnosis of multifactorial wear, primarily chemical and functional.

**Figure 7:**
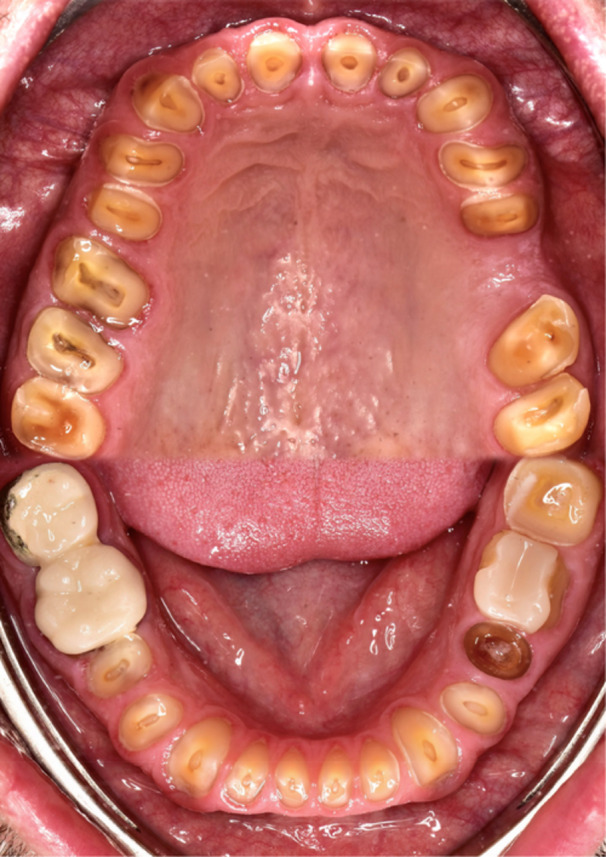
Intraoral occlusal photographs, showing clear signs of erosion, attrition, and abrasion, with near‐complete loss of clinical crown height in most of the teeth.

Intraoral examination showed advanced tooth wear in both anterior and posterior teeth. (Figure 7) without pulpal exposure or acute sensitivity, suggesting chronic adaptation. Four teeth (2.6, 2.7, 3.5, and 4.6) exhibited signs of endodontic pathology, requiring root canal treatment or extraction.

**Figure 8:**
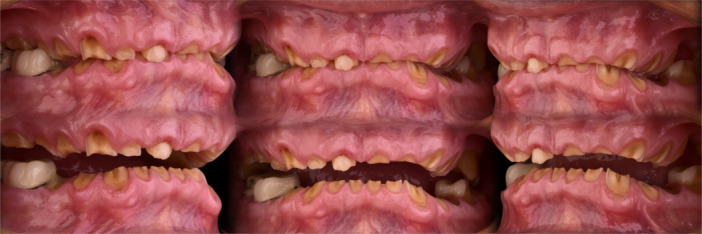
Functional evaluation revealed an edge‐to‐edge incisal relationship, in which both the upper and lower anterior teeth had worn into contact. In this context, any attempt to restore the incisal edges would necessitate an increase in the VDO.

The initial questionnaire, combined with clinical examination and CBCT imaging, ruled out TMD and sleep apnea risks, with normal pharyngeal airway dimensions. As sleep diagnostics were unnecessary, increasing VDO was deemed safe respiratory‐wise. The lack of ferrule (Figure 8) posed a biomechanical challenge, classifying the patient in the Yellow category, requiring pretreatment before prosthetic rehabilitation.

After confirming functional stability, the SDS concept was applied. Photographs showed no visible upper incisors at rest and minimal gingival display during smiling. Normally, 1–4 mm of maxillary incisor should be visible at rest, influenced by factors like lip length and age. Digital smile design indicated a need for clinical crown lengthening, with an estimated 6 mm increase at the right maxillary central incisor.

**Figure 9:**
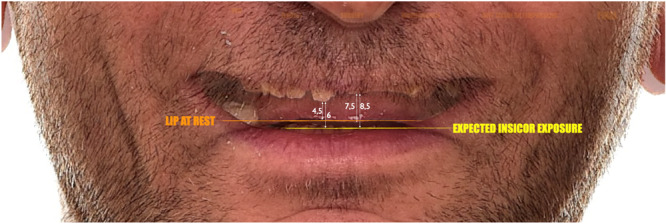
Using calibrated overlays on the patient's extraoral photographs, the desired position of the upper central incisors was determined to require approximately 6 mm of lengthening.

**Figure 10:**
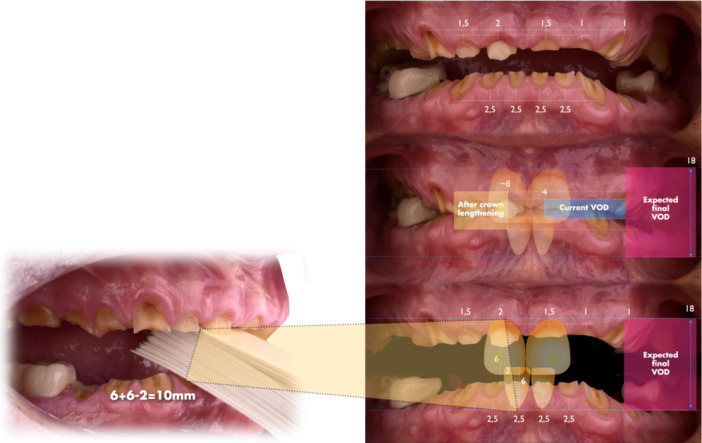
The planned 18 mm space aligned precisely with esthetic goals established through the Smile Design. The upper central incisors were to measure 11 mm, matching the 6 mm planned elongation. Mandibular incisors required a final length of 8–9 mm, achieved via 2.5 mm apical surgical extension and around 6 mm of prosthetic lengthening. A 2 mm vertical overbite was maintained to ensure esthetic and functional balance. With spatial relationships confirmed, the bite was registered at the new VDO (10 mm above baseline), enabling a preliminary wax‐up to assess restorative feasibility. For a patient with no TMD symptoms and a repeatable occlusion, registration of the new VDO is best performed using a leaf gauge.

A treatment plan was developed to increase the clinical crown length on the labial and palatal surfaces of the maxillary incisors for improved esthetics and adequate ferrule. The initial VDO was only 4 mm between opposing central incisors, well below the functional and esthetic norm (Figure 9). Post‐surgical crown lengthening was expected to increase this by approximately 4 mm, achieving an 8 mm separation. The “18 mm Rule” (Refenauct and Lee 1990) defines the typical vertical distance between the zenith of an 11 mm maxillary incisor and a 9 mm mandibular incisor as 18 mm, accounting for a minimal 2 mm vertical overbite to ensure proper incisal guidance. In the present case, the measured distance was significantly reduced due to severe tooth wear, and a further 10 mm increase in VDO was required to re‐establish functional and esthetic parameters (Figure 10).

**Figure 11:**
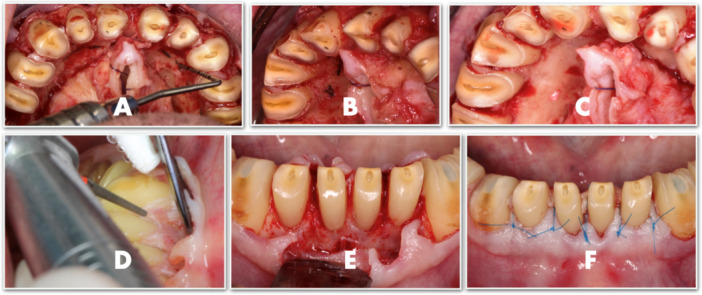
Crown lengthening in the maxillary arch involved both the buccal surfaces for esthetic purposes and the palatal surfaces to improve biomechanics (A–C). In the mandibular arch, crown lengthening was limited to the labial surfaces only, addressing both esthetic and biomechanical considerations (D–F).

Crown lengthening in this case aimed not only to improve esthetics but, more importantly, to recreate the ferrule, which is essential for proper biomechanical performance. The lack of palatal ferrule on the maxillary anterior teeth posed a significant biomechanical and functional challenge. Although the mandibular incisors exhibited approximately 2 mm of ferrule height, their pyramidal morphology offered limited resistance. To optimize conditions for shoulderless preparations and establish a stable supracrestal attachment in the maxilla, surgical crown lengthening was performed. This included full‐thickness flap elevation and osteogingivoplasty with both buccal and palatal bone reduction. Palatal flaps were selectively thinned to minimize postoperative soft tissue rebound, and surgical markings were placed 3 mm apically to facilitate ideal repositioning. Interdental papillae were preserved to maintain the existing esthetic gingival architecture (Figure 11).

This maxillary surgical phase formed part of a broader pretreatment protocol that unfolded over four stages. In the initial stage, crown lengthening was performed in the upper arch: buccal and palatal osteogingivoplasty was carried out on teeth 13–23 without papilla reduction, and circumferential crown lengthening was performed on teeth 14, 15, 24, and 25. Simultaneously, teeth 16, 17, and 46 were atraumatically extracted after root separation.

Subsequent surgical stages addressed the mandibular arch. Crown lengthening was carried out circumferentially on teeth 34, 35, 44, and 45, and on the buccal surfaces of teeth 31–33 and 41–43. Tooth 46, which had been sectioned earlier, was removed during this stage.

After adequate healing, implant placement was performed. At site 26, an implant (Astra Tech EV, Dentsply Sirona) was placed using a crestal sinus lift with the hydrodynamic Osstem CAS kit, along with a connective tissue graft harvested from the palate to augment soft tissue volume. A second implant was placed at site 46 (Astra Tech PrimeTaper). The final surgical session involved the placement of implants and healing abutments at sites 16 and 17 (Astra Tech EV, Dentsply Sirona), also using a crestal sinus lift approach.

**Figure 12:**
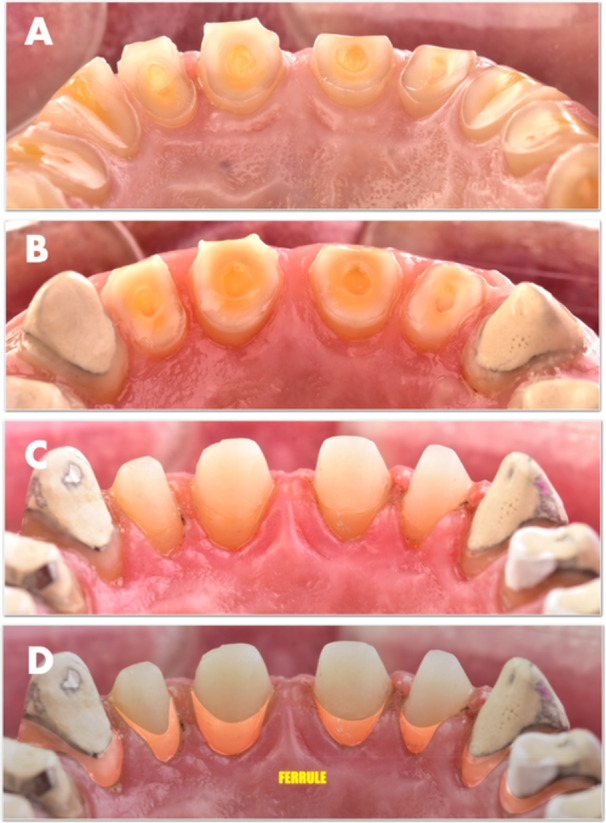
(A) Palatal ferrule of 2 mm recreated through surgical crown lengthening. (B) Canines and premolars restored using custom cast metal posts. (C) Incisors reconstructed with direct composite build‐ups. (D) The ferrule constitutes a significant portion of the core that will be covered by the final crown.

Following healing, root canal treatment was selectively performed only on teeth subjected to increased horizontal vector forces, such as canines and premolars. These were restored with opaque cement‐coated metal posts to enhance mechanical stability. In contrast, the anterior teeth, with restored ferrule and reduced anticipated load, were left vital and restored without posts. The presence of enamel at the preparation margins allowed for additional elongation using composite resin, resulting in functional outcomes while maximizing preservation of the remaining tooth structure. (Figure 12)

**Figure 13:**
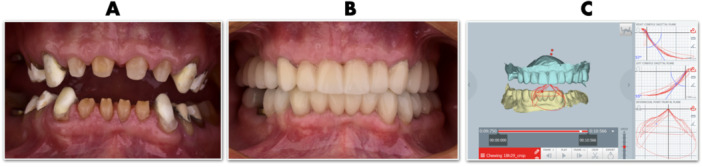
Following the reconstruction of the abutment teeth (A), provisional PMMA crowns were fabricated to guide soft tissue contouring (B) and to register the patient's chewing cycle at the new vertical dimension using Modjaw digital axiography (C).

**Figure 14:**
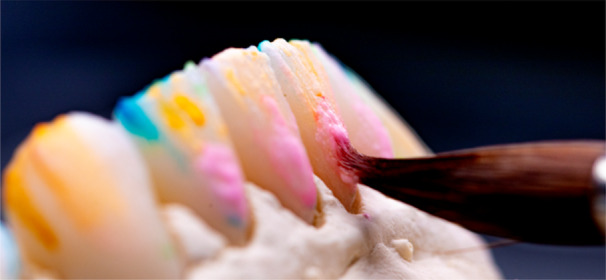
To enhance esthetics, the labial surfaces of the anterior crowns were additionally manually veneered with layered feldspathic ceramic.

After reconstructing the abutment teeth, provisional polymethyl methacrylate (PMMA) crowns were fabricated to guide soft tissue healing and evaluate esthetics, phonetics, and function. These temporaries were also used to register the patient's chewing cycle at the new vertical dimension using Modjaw digital axiography, (Figure 13), allowing for individualized planning of the occlusal surface morphology. The main objective was to establish equal, bilateral, simultaneous contact points with smooth functional guidance and no interferences during mastication. Knowing that the patient is a bruxer and will likely continue to protrude the mandible, it was crucial to ensure proper force distribution by establishing even eccentric contacts in the edge‐to‐edge position on as many anterior teeth as possible. The final crowns on four implants were milled from zirconia, while on natural teeth were created using a cut‐back technique with lithium disilicate (Figure 14).

**Figure 15:**
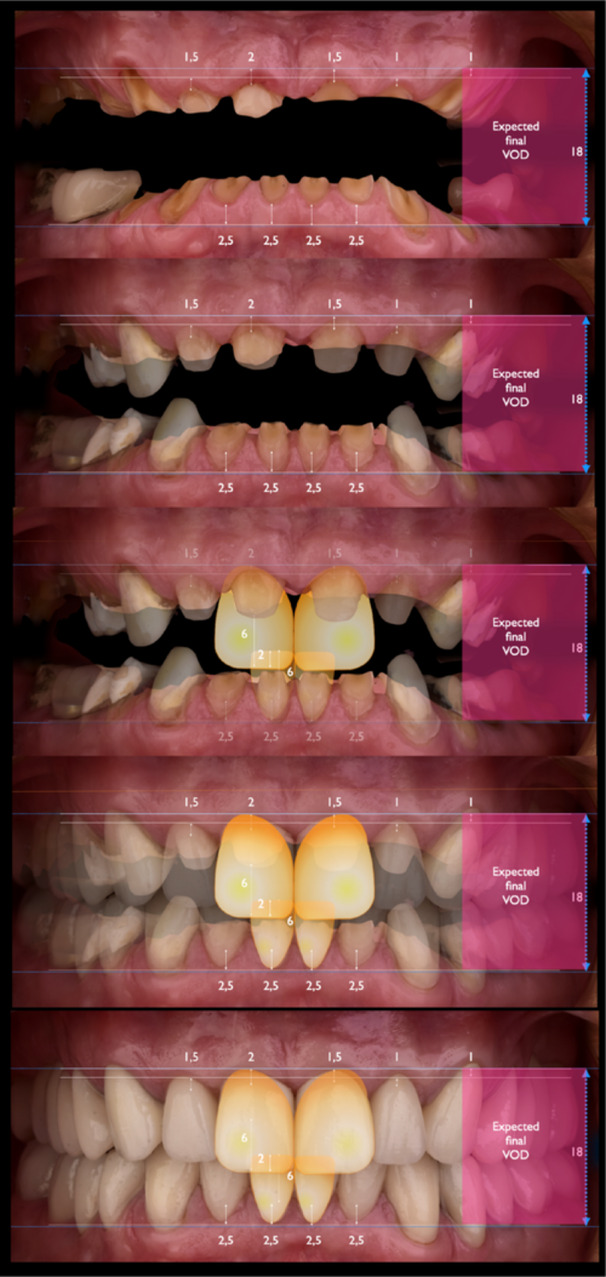
The treatment progression from the initial planning stage to the final stage.

**Figure 16:**
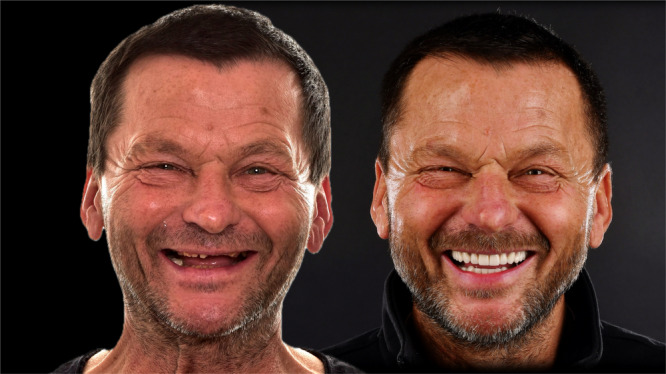
Extraoral photograph comparison showcasing improved esthetics after full mouth reconstruction.

**Figure 17:**
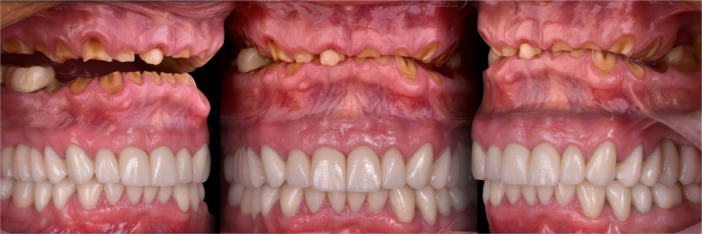
Intraoral view pre‐post comparison, showing improved occlusal relationships.

The treatment progression from the initial to the final stage is summarized in Figure 15. The esthetic outcome (Figure 16) and the occlusal and functional result (Figure 17) were highly satisfactory for the patient. One of the most important elements in the reconstruction of worn dentition is protecting the new restorations with a hard, flat night guard, which—like the teeth themselves—should provide at least one contact point per tooth and guidance that eliminates balancing interferences.

**Figure 18:**
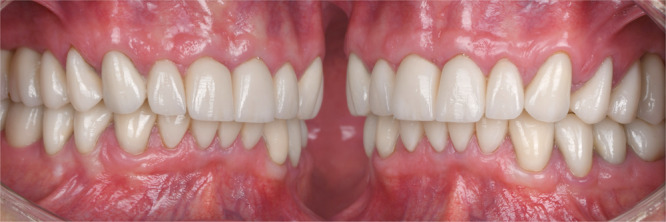
Two‐year follow‐up lateral intraoral photos, showing stability of the restorations and periodondal tissues, with no biological or technical complications observed.

At the 2‐year follow‐up, all restorations remained intact, providing favorable esthetics and function, with no biological or technical complications observed (Figure 18). The patient maintained excellent oral hygiene and consistently used a nightguard. He made significant lifestyle adjustments, including discontinuing prolonged fasting and vitamin C supplementation, reducing the risk of recurrence. The TMJs remained asymptomatic, and both esthetic and functional parameters were maintained, confirming the long‐term stability of the SDS‐guided treatment protocol.

## 4 Discussion

One of the most persistent clinical challenges in restorative dentistry has been increasing the VDO. For decades, clinicians have approached changes to VDO with caution, due to concerns about neuromuscular instability, TMJ dysfunction, or patient discomfort. However, a recent comprehensive review by Lassmann et al. suggests that many of these of concerns are either outdated or unsupported by current evidence (Lassmann, Calamita, and Manfredini 2025). The literature indicates that increases of up to 5 mm are safe and predictable in well‐diagnosed and properly managed cases (Abduo 2012). More importantly, there is no publication to date has demonstrated that VDO increases beyond 5 mm are inherently dangerous; such increases simply require individualized planning and careful monitoring.

In this case, a 10 mm increase in the VDO was planned using the SDS Concept, which provides a structured approach for assessing restorative space, incisal display, and occlusal function. By applying formulas such as A + B − C = VDO, the clinician was able to determine the exact space needed for restorative reconstruction while maintaining functional harmony and airway integrity. This method moves away from outdated facial proportions or removable prosthodontic measurements, providing a reproducible and esthetically driven planning model.

Another topic frequently debated in the literature is the restoration of structurally compromised teeth. Clinicians often focus on the choice between fiber versus metal and cast versus prefabricated posts, yet evidence consistently shows that establishing a proper ferrule is far more critical than the specific type of post used. The ferrule effect refers to the reinforcing benefit provided by a circumferential band of tooth structure, which helps resist fracture and dislodgement of a restored tooth by improving stress distribution—this applies to both endodontically and non‐endodontically treated teeth. For the ferrule effect to be effective, adequate dimensions are essential—minimum height of 2 mm (Samran et al. 2013) and thickness of 1.5 mm (Samran et al. 2025; Xie et al. 2020), minimal taper, and proper positioning. In maxillary teeth, due to the centrifugal direction of occlusal forces exerted by the mandibular teeth, the palatal ferrule is biomechanically most critical as it acts as a barrier against dislodging forces (Zhang et al. 2015; Santos Pantaleón et al. 2018; Jasim 2016). Although no specific studies define the optimal ferrule location for mandibular incisors, the labial ferrule is presumed to be key, considering the direction of acting forces. Once an adequate ferrule is achieved, the survival rates of various post types are comparable, and in some cases, a post may not be necessary at all (Lazari et al. 2018; Magne et al. 2017; Naumann et al. 2018; Tsintsadze et al. 2024).

The ongoing debate likely persists due to clinical biases, selective literature interpretation, and confusion between survival and success rates. While teeth lacking a ferrule restored with fiber posts may fracture more easily, but these failures are usually repairable. Conversely, teeth with a ferrule and a metal post are more fracture‐resistant, but their failures are often non‐restorable, leading to tooth loss (Bacchi et al. 2019). Therefore, the choice of post should be guided by clinical judgment, considering reparability versus durability. For example, preserving a maxillary central incisor in a young patient with a high smile line might favor a reparable solution over long‐term strength. Meanwhile, older bruxer (such as this patient) may benefit more from a rigid, highly retentive structure, accepting the risk of non‐restorable failure in favor of greater stability.

While establishing a ferrule is critical for mechanical stability, crown lengthening should be carefully considered in relation to the crown‐to‐root ratio (CRR). Meng et al. highlighted that a CRR greater than 1.0 can compromise a tooth's biomechanical performance, especially when the root structure narrows apically (Meng et al. 2023). Therefore, the ferrule design and supporting bone level must be evaluated together, especially when significant VDO increases are necessary. This aligns with finding from Ashnagar et al. who reported a 91% long‐term survival rate of teeth treated with crown lengthening, as long as supracrestal tissue height was respected and root support maintained. Their 7‐year retrospective analysis supports the clinical viability of crown lengthening when guided by sound biomechanical principles, including CRR assessment and preservation of supracrestal tissue attachment (Ashnagar et al. 2019).

Despite being based on a single case, this report illustrates how a dual‐algorithmic approach, first assessing functional risk (TMD, bite stability, interdisciplinary needs), and then applying the SDS concept, can yield a treatment plan that is biomechanically sound, esthetically satisfying, and functionally predictable. After 2 years, the increased vertical dimension remains stable, no TMJ symptoms have developed, and the restorative outcome continues to meet clinical and patient expectations. This supports the concept that individualized VDO increases, even beyond 5 mm, are not only possible but also safe when guided by logic and science evidence.

## 5 Conclusions

This clinical report demonstrates how combining a functional risk classification (Green, Yellow, Red) with the SDS Concept provides a structured, biologically sound, and esthetically guided framework for full‐mouth rehabilitation in cases of severe tooth wear. The SDS approach offers a precise, incisal‐display‐based method for adjusting vertical dimension while avoiding arbitrary standards. Incorporating symptom‐based questionnaires reveals hidden etiologies such as parafunction or systemic factors. This emphasis on ferrule recovery, repeatable occlusion, and repairable restorations ensures predictable biomechanics.

## Author Contributions

**Łukasz Lassmann:** conceptualization, project administration, data curation, writing – original draft, writing – review and editing. **Adam Pióro:** resources, data curation, methodology, writing – original draft. **Javier Calatrava:** supervision, methodology, writing – original draft, review and editing. **Hom‐Lay Wang:** supervision, critical review, and editing.

## Funding

The authors have nothing to report.

## Disclosure

All authors gave final approval and agreed to be accountable for all aspects of the manuscript.

## Conflicts of Interest

The authors declare no conflicts of interest.

## Data Availability

Study data are available from the corresponding author upon reasonable request.
